# An Emerging Facet of Diabetes Mellitus: The Nexus of Gastrointestinal Disorders

**DOI:** 10.7759/cureus.18245

**Published:** 2021-09-24

**Authors:** Srimy Modi, Naqvi Syed Gaggatur, Aliya H Sange, Natasha Srinivas, Mubashira K Sarnaik, Mohammad Hassan, Harini Gajjela, Ibrahim Sange

**Affiliations:** 1 Research, K.J. Somaiya Medical College, Mumbai, IND; 2 Internal Medicine, M S Ramaiah Medical College, Bangalore, IND; 3 Research, BGS Global Institute of Medical Sciences, Bangalore, IND; 4 Internal Medicine, Mohiuddin Islamic Medical College, Mirpur, PAK; 5 Research, Our Lady of Fatima University College of Medicine, Valenzuela, Metro Manila, PHL; 6 Research, California Institute of Behavioral Neurosciences and Psychology, Fairfield, USA

**Keywords:** celiac disease, non-alcoholic fatty liver disease, autoimmune gastritis, autonomic neuropathy, pro-kinetic agents, bariatric surgery, colorectal carcinoma, diabetic gastroparesis, gastrointestinal disorders, diabetes mellitus

## Abstract

Diabetes mellitus (DM) is a chronic metabolic disorder with a multi-systemic involvement, the gastrointestinal (GI) system being one of them. In this study, we have compiled and analyzed findings from various studies to conclude that peripheral insulin resistance and hyperglycemia are the two key factors that play a role in the pathogenesis of the web of disorders associated with diabetes. These two key factors, when clubbed with autoimmunity, autonomic neuropathy, and genetic and environmental factors, play a substantial role in the development of GI disorders in DM. This article examines GI disorders such as gastric autonomic neuropathy, non-alcoholic fatty liver disease (NAFLD), celiac disease (CD), etc. It also highlights the importance of regular screening and assessment of DM in preventing the GI tangent of the disease. A prompt blood glucose control through lifestyle modifications, dietary management, and weight reduction, coupled with pharmacotherapy for existing DM, can lead to a better outcome and an optimistic perspective on the disease.

## Introduction and background

Diabetes mellitus (DM) is an endocrine, metabolic disorder characterized by elevated blood glucose levels caused by limited insulin production or peripheral resistance to its actions [1]. It is categorized into four different subtypes depending on their etiopathogenesis, as presented below in Table 1 [1].

**Table 1:** Classification of diabetes mellitus based on etiopathogenesis

	Diabetes mellitus types	Etiopathogenesis
1.	Type I diabetes mellitus (T1DM)	Immune-mediated pancreatic beta-cell destruction
2.	Type II diabetes mellitus (T2DM)	Peripheral insulin resistance
3.	Gestational diabetes mellitus (GDM)	Pregnancy-induced glucose intolerance
4.	Others	Chemical drugs, pancreatic and genetic disorders

DM is one of the quiescent causes of various chronic systemic diseases that result in significant public health burdens worldwide [2]. According to the International Diabetes Federation's 2017 edition of IDF Global Atlas, it was estimated that around 425 million people were affected by diabetes worldwide [2]. And this number was expected to rise up to 700 million by 2045 [2]. Insulin resistance in the muscles is caused by the decreased recruitment of the glucose transporter 4 (GLUT-4) proteins to the plasma membrane in response to insulin [3]. As a result, decreased cellular uptake and breakdown of glucose lead to the elevation of extracellular glucose concentration [3]. Reduced glycogen storage in the liver further aggravates this condition [3]. During fasting states, there is uncontrolled gluconeogenesis in the liver due to the failure of insulin-mediated suppression [4]. Insulin resistance at the level of fatty tissues leads to reduced insulin-mediated glucose uptake for conversion into fats, and increased lipid breakdown, with an elevation of the free fatty acids (FFAs) and cytokines, leading to a state of chronic inflammation [5].

Uncontrolled diabetes can be complicated with diabetic ketoacidosis or hyperosmolar hyperglycemic non-ketotic coma even without any prior history of diabetic manifestations [6]. However, the classical features that help make a provisional diagnosis include polyuria, excessive thirst, fatigue, dehydration, vomiting, and altered mental status [6]. Patients presenting with symptoms mentioned above can be diagnosed with diabetes based on the presence of any of the following parameters: random plasma glucose of >200 mg/dl or glycosylated hemoglobin (HbA1c) levels >6.5% or fasting plasma glucose level >126 mg/dl. Other tests include an oral glucose tolerance test (OGTT) with a 75 gm glucose load with a two-hour postprandial (PP) plasma glucose level of >200 mg/dl [7].

One of the quintessential steps to manage diabetes is through comprehensive lifestyle management, which includes weight management and physical activity, as it is beneficial in controlling blood glucose levels and blood pressure [8]. Treatment of diabetes varies among patients based on specific characteristics like age, weight, lifestyle, personal preferences, stage of disease, compliance, and any other comorbid conditions [9]. As per clinical trials, the first-line treatment of diabetes is metformin, but a few studies have shown that oral or parenteral hypoglycemic agents are an alternative in case of adverse drug reactions (ADRs) [9].

Hyperglycemia in diabetes leads to the formation of advanced glycation end products (AGEs). The AGEs lead to free radical injury and biochemical alterations in the eyes, nerves, and vascular and renal tissues, leading to diabetic retinopathy, cataract, atherosclerosis, neuropathy, nephropathy, etc. [10].

It is critical to delve into the GI complications associated with diabetes as they are not extensively explored. Some substantial complications in DM include delayed gastric emptying (GE), esophageal motility disorder, chronic diarrhea, gastroesophageal reflux disease (GERD), non-alcoholic fatty liver disease (NAFLD), etc. [11]. The common GI symptoms include nausea, vomiting, satiety, flatulence, gastric fullness, abdominal pain, etc. Achieving reasonable glycemic control through various lifestyle and pharmacological approaches is imperative in managing the mentioned complications [11]. In light of these factors, this review article aims to:

1. Discuss the spectrum of GI complications associated with diabetes.

2. Explore the proportionality between poorly controlled diabetes and the severity of GI symptoms.

3. Emphasize the importance of early diagnosis and management of diabetes to prevent the occurrence of GI complications.

## Review

Irrespective of the age group, DM can manifest as a multi-systemic condition due to chronic inflammation, vascular endothelial damage, organic and functional lesions, etc. When left uncontrolled for extended periods, DM can cause many devastating disorders involving any part of the alimentary tract [12].

Gastrointestinal autonomic neuropathy in diabetes

**Figure 1:**
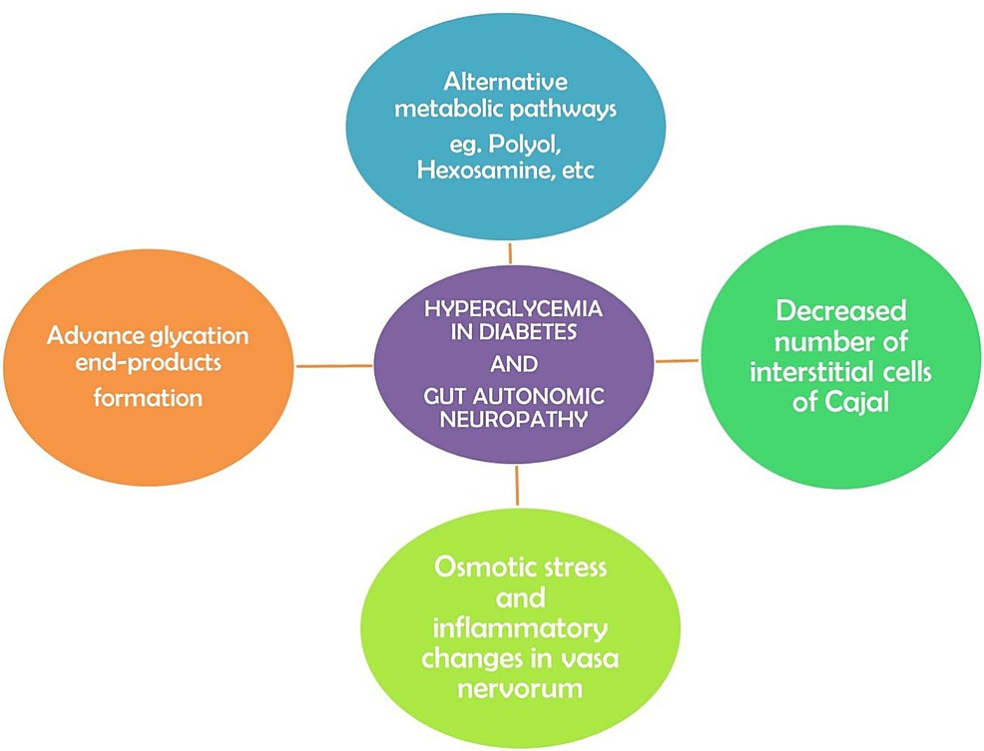
Summary of factors involved in the pathogenesis of gastrointestinal autonomic neuropathy in diabetes

About 100 million meticulously organized neurons in the enteric nervous system regulate the motility of the gut via the myenteric nerve plexus, and the absorption and secretion by the submucous network [13]. The interstitial cells of Cajal (ICC) act as pacemakers and messengers for carrying impulses from the nerves to the smooth muscles [13]. DM can chronically disrupt the GI tract's enteric, autonomic, and somatic nervous systems [13]. Hyperglycemia diverts the excess glucose molecules into alternative metabolic pathways like polyol, hexosamine, etc. [14]. The glucose molecules attach to the fats or proteins and lead to the formation of the AGEs [14]. AGEs lead to free radical injury and oxidative damage to the nerves and deteriorate the nerve structure and function [15]. There is a decrease in the number of ICC, damage to the smooth muscle cells, and central nervous system (CNS) neurons, which cause a contractile dysfunction of the gut [15]. Hyperglycemia creates osmotic stress and inflammatory changes and destroys vasa nervorum, the small vessels supplying the nerves, contributing further to the neuropathy occurring in diabetes (Figure 1) [15].

Bharucha et al. conducted a study in 2015 on 78 type 1 diabetic patients, which assessed the patients with 13C-spirulina breath test for GE and GI symptoms associated with diabetes. A variable result was obtained, such that 50% (37/78) of the participants had a normal GE, 47% (35/78) had a slow GE, and only 3% (2/78) had a rapid GE. Thus, the study concluded that in T1DM patients, GE was delayed and strongly associated with the GI symptoms and the early or long-duration hyperglycemia (Table 2) [16]. Frøkjær et al. conducted a case-control study on 12 type 1 diabetic patients with GI manifestations and autonomic neuropathy against 12 healthy controls by using a specially designed ultrasound probe to measure the evoked duodenal and esophageal contractile activities. The T1DM subjects had a raised frequency of distention-evoked contractions (6.0 ± 0.6 vs. 3.3 ± 0.5, p<0.001) that correlated with the duration of the disease (p=0.009). The study concluded that an abnormal contractile response is a reflection of neuronal abnormalities of the gastrointestinal tract (GIT) due to diabetic autonomic neuropathy (Table 2) [17]. Diabetes can present with variable GI symptoms such as nausea, vomiting, abdominal pain, GERD, abnormal bowel movements, etc. [15]. A questionnaire-based study conducted by Bytzer et al. in 2001 on a group of 15,000 subjects concluded that the patients with poorly controlled DM, irrespective of the duration of diabetes or the ongoing treatment, had a higher prevalence of upper and lower GI symptoms [18]. 

The GI manifestations in DM occur due to the unbalanced digestive functions of the proximal part of the GIT, and correlate with abnormal retention of food in the upper GIT, without any evidence of mechanical obstruction [19]. This GI spectrum of diabetes can be described simply by gastroparesis, a syndrome wherein the delayed GE leads to backward pressure and upper GI manifestations, especially PP fullness. However, the definition of gastroparesis is not strictly adherent to a particular set of GI symptoms and is rather complex and inconsistent [19,20]. The weakened esophageal contractions and lower esophageal sphincter (LES) tone can lead to GERD and dysphagia in diabetic patients [21]. Chronic GERD is a common complaint in poorly controlled diabetics with comorbid risk factors like age, male sex, obesity, sedentary lifestyle, etc. [21]. Chronic and long-duration GERD could progress to metaplasia of the lower one-third of the esophagus known as Barrett's esophagus [21]. The sequelae to Barrett's esophagus is adenocarcinoma of the esophagus [21].

**Figure 2:**
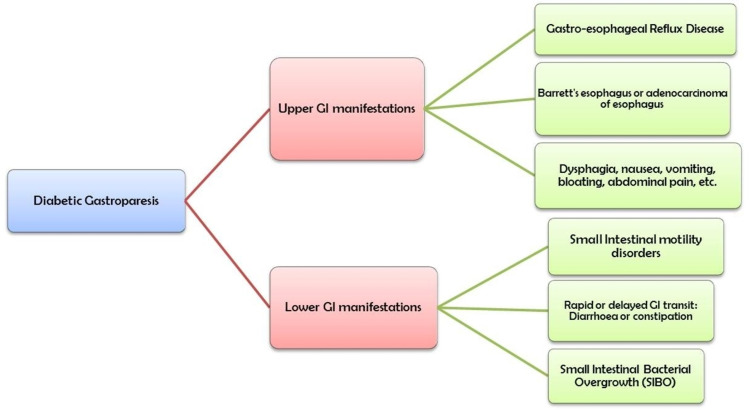
Upper and lower GI manifestations associated with diabetes mellitus GI: gastrointestinal; SIBO: small intestinal bacterial overgrowth

Alterations in small intestinal motility can present as a delayed or rapid food transition through the gut [22]. While quick passage of food contents causes a condition called diabetic diarrhea, a delayed transit causes accumulation of food within the small intestine leading to small intestinal bacterial overgrowth (SIBO) (Figure 2) [22]. SIBO is a condition of imbalance in the gut microbiome, and an increase in the small intestinal bacterial count [23]. Any pathology leading to a stagnation of intestinal contents can contribute to SIBO, and autonomic neuropathy in diabetes is one of them [23]. The most commonly used tests to diagnose SIBO are methane or hydrogen breath tests, but the gold standard investigation is a jejunal aspiration to assess the bacterial count [23].

Commonly used diagnostic tests to assess gastroparesis in diabetes include isotopic scintigraphy [12]. More than 60% of the radioactivity after two hours and 10% of the radioactivity after four hours of the test is conclusive [12]. Another non-invasive but more expensive method of assessment includes external electrogastrography [12]. Bharucha et al. conducted a study in 2008 on 129 patients with an approximately equal number of type 1 and 2 diabetics. According to the survey, 36% of the sample population had delayed GE measured through scintigraphy, whereas 22% had a rapid GE, concluding that diabetes is associated with a prompt and slow GE (Table 2) [24]. Choung et al. conducted a community-based study on diabetic patients in Olmsted County, Minnesota, to establish the relationship between DM and the risk of developing gastroparesis. The study utilized isotopic scintigraphy and symptom-based analysis to assess the presence of delayed GE. It concluded that the risk of developing delayed GE was higher in type 1 diabetes compared with type 2 diabetes (Table 2) [25].

Lifestyle modifications and pharmacological treatment are the primary modes of diabetes management [12]. However, the treatment highly depends on the patient compliance and disease severity [12]. Dietary modifications are advised more frequently and are highly efficacious in alleviating the GI symptoms [12]. It includes intake of small meals at frequent intervals and a preference for a low-fat diet [12]. The first-line treatment of the GI complications includes pro-kinetic agents, such as itopride, metoclopramide, ghrelin agonists like relamorelin, etc. [12]. Studies have shown that an artificial glucose infusion can elevate blood glucose levels leading to delayed GE time [26]. Likewise, insulin administration and induction of hypoglycemia can lead to a rapid GI emptying of the concrete and liquid feeds [26]. Thus, an achievement of a reasonable glycemic control through oral hypoglycemic agents (OHAs), insulin administration, or dietary management would alleviate the GI symptoms in diabetes [26]. Bytzer et al. conducted a cross-sectional questionnaire-based study in a group of 1,101 subjects with DM. The study found that upper GI symptoms independently depended on the self-reported glycemic control and HbA1C levels rather than the duration or type of diabetes. The study concluded that poor control of diabetes is associated with a higher incidence of GI complications in diabetics [27].

**Table 2:** Summary of studies performed on diabetic patients for the assessment of gastric emptying GE: gastric emptying; GI: gastrointestinal; DM: diabetes mellitus

Reference	Study design	Cases of diabetes	Observation	Study population	Methods used	Conclusion
Bharucha et al. (2015) [16]	Randomized controlled trial	78 patients	GE – average: 37 (50%), delayed: 35 (47%), rapid: 2 (3%)	Participants with type 1 diabetes	Gastric emptying assessed by ^1^^3^C-spirulina breath test	Early and long-term hyperglycemia was strongly associated with delayed GE and GI symptoms
Frøkjær et al. (2007) [17]	Case-control study	Type 1 diabetics: 12, healthy controls: 12	Type 1 diabetics: increased frequency of distension-induced contractions (6.0 ± 0.6) vs. (3.3 ± 0.5) p<0.001		Esophageal and duodenal contractions were measured using bag distension and ultrasound probe	An impaired contractile activity in type 1 diabetes patients due to autonomic neuropathy
Bharucha et al. (2009) [24]	Comparative analysis	129 patients	GE – average: 55 (42%), delayed: 46 (36%), rapid: 28 (22%)		GE transit assessed by scintigraphy	Rapid and slow GE is associated with diabetes
Choung et al. (2011) [25]	Prospective, cohort study	Type 1 DM: 227, type 2 DM: 360, controls (age and sex-matched non-diabetics): 639	Cumulative proportion of developing gastroparesis in 10 years – type 1 DM: 5.2%, type 2 DM: 1.0%, controls: 0.2%	A resident population of Olmsted County	Cox proportional hazard modeling	Increased risk of gastroparesis in type 1 diabetes, but otherwise uncommon

Type 1 diabetes and celiac disease

A strong tie that binds type 1 diabetes and celiac disease (CD) depends on a complex interplay of genetic and environmental factors. An Italian multicenter study conducted on children and adolescents with T1DM screened the subjects for CD. The study found a higher prevalence of biopsy-proven CD in the subjects [28]. Human leukocyte antigen (HLA)-DQ2 positivity explains the higher prevalence of CD in T1DM [29]. HLA-DR3/DQ2 is strongly expressed in CD (more than 90%) and T1DM (around 55%) but only up to 25% in the general population [29].

T1DM patients that are genetically predisposed, on exposure to specific environmental triggers such as viral infections, milk protein, gluten proteins, or certain toxic compounds like nitrosamines, can develop CD [30]. T1DM and CD tend to occur together due to the common autoimmune mechanism, characterized by inflammation and antibody-mediated destruction of the tissues [31]. Immune dragged destruction of insulin-producing pancreatic beta (β) cells is the leading cause of T1DM, causing a state of hyperglycemia [31]. Similarly, gluten intake from wheat, barley, or rye can trigger an immune-mediated attack over the small intestinal mucosa characterized by villous atrophy, lymphocytic infiltration, and cryptic hyperplasia in CD [31]. CD most commonly presents with GI symptoms such as malabsorption leading to malnutrition, diarrhea, constipation, vomiting, abdominal pain, wind, or failure to thrive in children below three years [32]. It can also present with non-GI manifestations in older children, such as fatigue, vitamin deficiencies, iron deficiency anemia (IDA), delayed puberty, short stature, etc. [32]. Even though GI manifestations may be present in a few T1DM patients with CD, there is a higher chance of an asymptomatic/silent expression of CD in T1DM [32]. Therefore, screening for CD in T1DM is of utmost importance [32].

**Figure 3:**
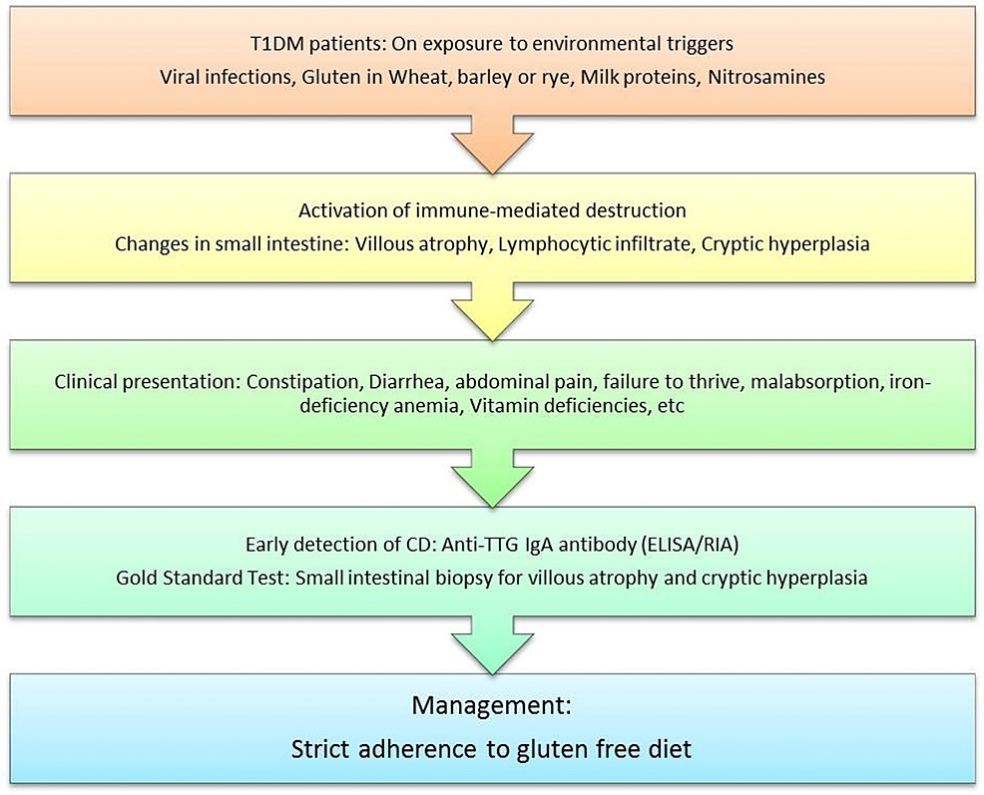
The proposed pathogenesis, screening methods, and management protocol for celiac disease in diabetes mellitus T1DM: type 1 diabetes mellitus; CD: celiac disease; anti-TTG IgA: anti-tissue transglutaminase immunoglobulin A

The single-most preferred test used to detect CD is enzyme-linked immunosorbent assay (ELISA) based detection of immunoglobulin A (IgA) anti-tissue transglutaminase antibody (TTG IgA), which has a high sensitivity (93%) and specificity (95%) [33]. Another serological test that is highly specific for CD is IgA anti-endomysial antibody (EMA) [33]. EMA is highly time-consuming, as it requires an immunofluorescence technique, and therefore less frequently utilized [33]. Serological detection is not sufficient to diagnose CD, and a definitive diagnosis is advisable [33]. The gold standard investigation for CD is a small intestinal biopsy [33]. Biopsies are taken from multiple sites of the duodenum, as CD causes a patchy involvement of the intestinal mucosa [33]. The presence of villous atrophy, cryptic hyperplasia, and elongated crypts is diagnostic of CD (Figure 3) [33]. Studies have shown that introducing a diet containing gluten in infants less than three months or more than six months of age is associated with a higher incidence of T1DM [34]. The most effective management of CD involves strict adherence to a gluten-free diet, as it can lower the incidence of T1DM and reduce complications associated with CD [34].

Non-alcoholic fatty liver disease in diabetes

**Figure 4:**
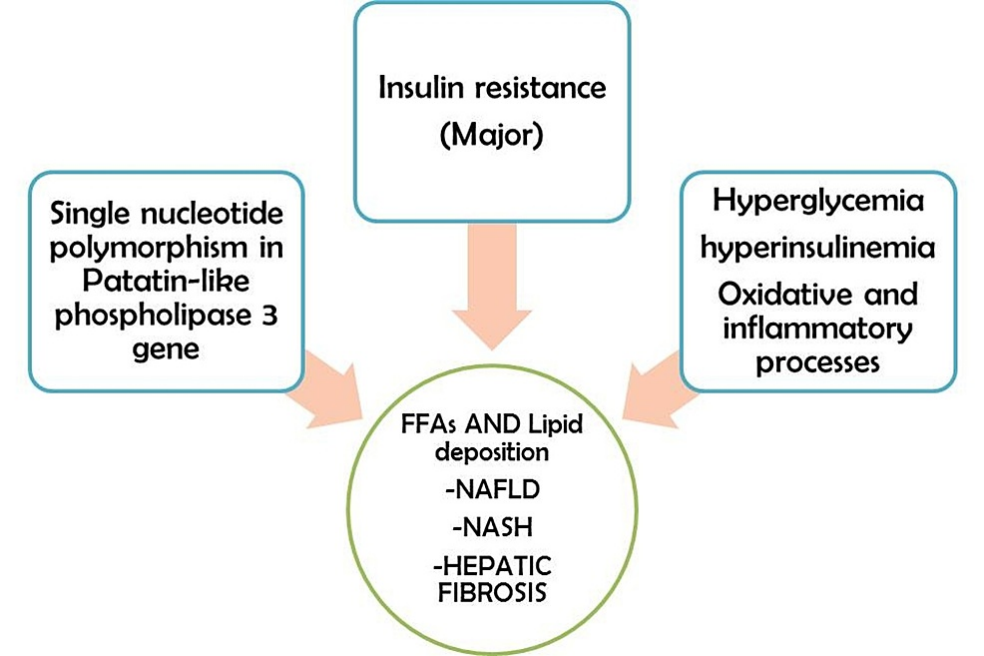
The underlying multifactorial pathogenesis of non-alcoholic fatty liver disease in diabetes FFAs: free fatty acids; NAFLD: non-alcoholic fatty liver disease; NASH: non-alcoholic steatohepatitis

In Western nations, NAFLD, also known as hepatic steatosis, is the most common liver disease and affects around 33% of the general population and 75% of obese patients with insulin resistance [35]. The pathophysiology that lies behind the co-occurrence of NAFLD and T2DM is complex and multifactorial [36]. Insulin resistance is the primary mechanism that causes the development of NAFLD [36]. However, emerging evidence shows that NAFLD could also occur in the absence of insulin resistance in cases of single nucleotide polymorphisms in the patatin-like phospholipase-3 enzyme-producing genes [36]. Three key sources that cause deposition of triacylglycerols (TAGs) in the liver are circulating FFAs (59%), de-novo lipid synthesis (26%), and dietary intake of fats (14%) [37]. Elevated serum glucose and insulin levels stimulate an inflammatory process within the liver that can further progress to hepatic steatosis [37]. Simple hepatic steatosis under the effect of the oxidative process, inflammatory cytokines, and mitochondrial dysfunctions can develop into full-blown non-alcoholic steatohepatitis (NASH), progressing to hepatic fibrosis (Figure 4) [37].

Lédinghen et al. recruited a group of 277 hospitalized diabetic patients in France in a cross-sectional study and followed them up with Fibrotest scoring and FibroScan. The results of the study suggested that 15.5% of the study population had severe liver fibrosis, especially elderly diabetics with a previous history of diabetic foot ulcers (Table 3) [38]. Cipponeri et al. in an Italian-based cross-sectional study included 220 type 1 diabetic adults who were not taking any vitamin D or calcium medications, and the study concluded that NAFLD prevalence was 29.5% and showed no association with vitamin D or calcium levels in the blood (Table 3) [39].

Addressing the metabolic risk factors through lifestyle modifications and weight control measures is the primary approach to managing NAFLD, as no medication is licensed for its treatment currently [40]. Interventional studies have shown that a short duration of the restricted calorie intake of around 28 days and exercise help improve hepatic steatosis adequately [40]. Vilar-Gomez et al. conducted a prospective interventional study involving 293 patients with histologically proven NASH. The subjects took a calorie-restricted diet for 52 weeks. The liver biopsies taken before and after the intervention were analyzed. The study concluded that weight loss was an effective intervention in the resolution of hepatic steatosis, as 25% of the study population achieved resolution while 19% showed fibrosis regression (Table 3) [41]. Bariatric surgery is another effective modality to treat obesity in diabetic patients [42]. It helps achieve optimal glycemic control in diabetes by causing weight loss and minimizes histological signs in NAFLD [42]. These effects are mainly due to postoperative weight loss, although it could also be due to incretin, which regulates blood glucose levels [42]. Xourafas et al. conducted a study in a subset of 756 patients undergoing bariatric surgery via Roux-en-Y gastric bypass (RYGB) or laparoscopic adjustable gastric banding (LAGB). The study demonstrated the effect of the surgery on the overall achievement of metabolic control and weight reduction in T2DM patients, which strongly correlated with normalization of alanine aminotransferase (ALT) and a decrease in HbA1C levels following the surgery (Table 3) [43].

**Table 3:** A detailed summary of studies that have assessed the prevalence of non-alcoholic fatty liver disease in diabetes mellitus T1DM: type 1 diabetes mellitus; T2DM: type 2 diabetes mellitus; NAFLD: non-alcoholic fatty liver disease; NASH: non-alcoholic steatohepatitis; ALT: alanine transaminase; HbA1C: glycosylated hemoglobin; RYGB: Roux-en-Y gastric bypass; LAGB: laparoscopic adjustable gastric banding

Author and year of study	Study design	Population studied, country	Sample (n), type 1/type 2	Diagnostic methods	Results	NAFLD prevalence	Conclusion
Lédinghen et al. (2012) [38]	Cross-sectional, prospective	Hospitalized diabetic patients, France	277, T1DM: 52%	Fibrotest scoring, FibroScan	Median Fibrotest score: 0.31, median liver stiffness: 4.8 kPA	Severe fibrosis: 15.5%	Higher prevalence of severe liver fibrosis in the study population, T2DM>T1DM
Cipponeri et al. (2019) [39]	Cross-sectional, prospective	Type 1 diabetic adults, Italy	220	Liver ultrasound		NAFLD positive: 57/220, grade 1: 51/57, grade 2: 5/57, grade 3: 1/57	NAFLD prevalence was higher in patients with diabetes irrespective of their vitamin D status
Vilar-Gomez et al. (2015) [41]	Interventional, prospective	Patients with histologically proven NASH, Cuba	293	Liver biopsies	Complete resolution: 72 (25%), reduction in NASH score: 138 (47%), regression of fibrosis: 56 (19%)		Weight loss and lifestyle interventions are associated with a resolution of NAFLD changes
Xourafas et al. (2021) [43]	Interventional, prospective	Patients recruited for bariatric surgery, Israel	756	Postoperative alanine transaminase (ALT) and HbA1C levels	Reduction in ALT post-RYGB: 20%, post-LAGB: 17%		ALT and HbA1C levels normalized in diabetics after bariatric surgery

Other GI diseases in diabetes

The spectrum of GI manifestations in diabetes is far-reaching and presents in multitudinous forms. The autoimmune destruction in T1DM is not confined to pancreatic β cells, but can further balloon out into a compilation of multi-systemic involvement called autoimmune polyglandular syndrome (AIPGS) [44]. Autoimmune gastritis (AIG) is an atrophic condition of the gastric fundal mucosa with the autoantibodies directed against the parietal cells (PCA) that secrete intrinsic factor (IF) [44]. The functional disruption of the parietal cells results in an exceptional form of anemia called pernicious anemia (PA) [44]. As compared to the general population, the prevalence of AIG and PA is around three to five times more in patients with T1DM, ranging from 5 to 10% and 2 to 4% respectively [44]. AIG is characterized by an assorted clinical presentation such as iron deficiency anemia with symptoms like fatigue, pallor, and reduced exercise tolerance [45]. Pernicious anemia is marked by a decreased absorption of vitamin B12 manifesting as macrocytic anemia and peripheral neuropathy [45]. In around 10% of patients, AIG can be considered as a susceptible cause for gastric carcinoid tumors or adenocarcinoma of the stomach [45]. Diagnosis of AIG can be made by gastric endoscopy, based on the presence of a shiny, red, and atrophic mucosal lining of the gut wall, and the absence of gastric rugae [44]. Characteristic biopsy findings in AIG include lymphocytic infiltration of the GI submucosa and lamina propria that can further progress to intestinal metaplasia and gastric adenocarcinoma [44]. Although the management guidelines are not distinct, it seems advisable to test the newly diagnosed T1DM patients for the presence of anti-parietal cell antibodies and the blood levels of gastrin, vitamin B12, and iron for the presence of anemia [44].

Recently, the fascinating association between colorectal carcinoma (CRC) and T2DM has attracted attention as there is a higher prevalence of both diseases, and it prompts us to lay further emphasis on counseling T2DM patients for the need to screen for CRC [46]. Insulin and insulin-like growth factor (IGF-1) play a crucial role in regulating cellular growth and proliferation [46]. Insulin effects are usually short-lasting and PP, but IGF-1 levels have a longer-lasting influence over the growth and proliferation [46]. Supra-physiologic levels of insulin cause a pro-proliferative effect over the colonic epithelium through cognate insulin and IGF-1 receptors [46]. Although IGF-1 and insulin are not mutagenic, the colonic epithelial changes lead to spontaneous mutations and follow the adenoma-carcinoma sequence, first mentioned by Fearon and Vogelstein [46]. Inculcation of cost-effective modalities for screening of CRC is cardinal to effective management of diabetic patients [46]. This can be achieved by counseling diabetes patients about the increased probability of developing CRC and advising screening techniques for an early diagnosis [46]. Fecal occult blood test (FOBT) is one of the effective screening techniques, as some of the adenomas and carcinomas tend to bleed into the GI tract and are visible as occult blood in the stool [46]. However, colonoscopy is the gold standard investigation in detecting CRC, with the highest sensitivity (96.7% for carcinomas) and specificity [46]. All the parts of the colon can be visualized through colonoscopy and the lesions can be excised and biopsied for histological analysis [46].

Pancreatic exocrine insufficiency is closely associated with T1DM due to common autoimmune pathogenesis leading to a deterioration of pancreatic exocrine and endocrine functions and insulin deficiency [47]. On the contrary, pancreatic exocrine insufficiency in T2DM is mediated by autonomic neuropathy and microvascular complications [47]. The customary symptoms of exocrine insufficiency include diarrhea, steatorrhea, failure to thrive in children, or pain in the abdomen [48]. Assessing the pancreatic exocrine function is tedious and can be done by direct or indirect methods [48]. Direct hormone-stimulated pancreas function test is a gold standard investigation to detect pancreatic exocrine insufficiency [49]. The principle of the test is to stimulate pancreatic enzymes by hormones administered exogenously [49]. Concomitantly performed duodenal intubation collects pancreatic secretions for assessment [49]. This test is highly sensitive and specific but has a slender clinical advantage due to its invasive nature and lower cost efficiency [49]. The indirect method includes fecal elastase-1 stool test and is common in practice, as it is comparatively cost-effective, unaffected by diet or fasting status, or simultaneous enzyme supplementation [49]. Fecal elastase-1 levels of less than 200 µg/g stool and less than 100 µg/g stool are suggestive of mild and severe pancreatic exocrine insufficiency, respectively [49]. The intake of smaller-sized and frequent meals is recommended in pancreatic exocrine insufficiency, but the foundation of treatment is an exogenous replacement of pancreatic enzymes [49].

Limitations

Although there is significant evidence that the GI spectrum of disorders occurring in diabetes is due to their shared roots of pathogenesis, there are also a few confounding variables that could indirectly be linked with the co-occurrence of GI diseases in DM. DM is a composite disease with a nexus of factors interlinking it with the GI system. Our report is limited and does not discuss the various GI complications occurring due to OHAs and other medications utilized in the treatment of diabetic patients. Supplementary research and detailed analysis are required to establish this association further.

## Conclusions

For years, countless studies have exhaustively assessed diabetic patients for GI disorders and upheld the association. In this review, we have compiled data from various other research articles that give us a clear view of the higher incidence and prevalence of GI-related symptoms in diabetic patients. This article has comprehensively discussed the relationship between NAFLD and diabetes due to abnormal lipid metabolism and oxidative liver damage. Furthermore, this paper has effectively incorporated data extracted from various studies to shed light on the role of genetics and autoimmune mechanisms in the development of certain autoimmune GI disorders with T1DM. Additionally, this article has accentuated the need to do an early screening of diabetic patients to prevent the development of GI complications. In conclusion, effective control of blood glucose levels through weight reduction, lifestyle modifications, and early diagnosis and management can lower the prevalence of specific GI manifestations. This study has elucidated the GI aspects of DM through a clear canvas of their interrelationships. However, there is a need to do an in-depth analysis to complement the validity of this association.
